# Immune ageing in skeletal fragility: clonal haematopoiesis and failure of inflammatory resolution during fracture repair

**DOI:** 10.3389/fimmu.2026.1913480

**Published:** 2026-08-17

**Authors:** Yong He, Hong-bin Xu, Jin Huang, Tian-peng Liu, Hai-feng Jia, Yi Shen, Fu-rui Fu, Jie Wang, Meng-ting Yuan, Xin Wei, De-zhi Tang, Xiang Gao

**Affiliations:** 1 Longhua Hospital, Shanghai University of Traditional Chinese Medicine, Shanghai, China; 2 Huadong Hospital, Fudan University, Shanghai, China

**Keywords:** cellular senescence, clonal haematopoiesis, fracture repair, immune ageing, inflammaging, inflammatory resolution, macrophages, osteoimmunology

## Abstract

Older adults with comparable bone mineral density or fracture-risk estimates may nevertheless differ markedly in fracture occurrence, repair kinetics and functional recovery. This heterogeneity suggests that skeletal fragility reflects, at least in part, ageing of the bone–immune–haematopoietic system rather than bone mass alone. Here, we synthesise evidence linking the aged osteoimmune niche to inflammatory bone loss and impaired fracture repair. We focus on four interrelated processes: CHIP-associated amplification of myeloid inflammation, shaped by driver mutation and clone size; age-related skewing of T- and B-cell compartments; persistent cellular senescence and senescence-associated secretory signalling; and failure of macrophage- and regulatory T-cell-mediated inflammatory resolution. Among CHIP drivers, direct skeletal evidence is strongest for DNMT3A-mutant clones in inflammatory bone loss. Bone-specific evidence for TET2 remains limited, whereas evidence for ASXL1 is largely absent. In fracture repair, the most direct preclinical evidence concerns cellular senescence and age-altered macrophage function, while the ability of CHIP to predict delayed union has not been validated. We define immune resilience as the capacity to mount a proportionate acute response, clear damaged cells and debris, actively resolve inflammation, and transition to reparative and remodelling states. Current evidence is insufficient to support routine immune-guided fracture care. The immediate priority is to determine, in prospective fracture cohorts, whether phase-resolved local and circulating measures explain variation in repair beyond bone mineral density, frailty, fracture characteristics, biological sex and treatment exposure.

## 1 Introduction

Clinical fracture prevention in later life has traditionally been anchored in BMD. Dual-energy X-ray absorptiometry (DXA)-derived BMD remains central to the diagnosis of osteoporosis and fracture-risk assessment, yet low-trauma fragility fractures are not restricted to individuals whose BMD lies in the osteoporotic range. Early cohort studies and later analyses showed that a substantial proportion of fractures occur in people with osteopenia. Because osteopenia is considerably more prevalent than osteoporosis, much of the population-level fracture burden arises from a broader group at moderate risk, rather than solely from the smaller subgroup at highest risk (1–4).

Risk-prediction tools such as FRAX have extended BMD-based assessment by incorporating clinical risk factors, including age, prior fracture, glucocorticoid exposure, smoking, alcohol intake and rheumatoid arthritis; FRAX can be calculated with or without BMD (5). A prior fracture confers a substantial increase in subsequent fracture risk beyond that explained by BMD (6). However, clinical-factor-based estimates do not directly measure bone microarchitecture or material properties, which require dedicated imaging or material-testing approaches (7–9), and routine assessment does not capture bone marrow niche biology or tissue-repair capacity. After a low-trauma fracture, imminent refracture risk rises, and hip fractures and other major osteoporotic fractures are associated with excess mortality (10–13). These outcomes reflect both the fracture event and the diminished physiological reserve of the population in whom it occurs; the epidemiological associations do not establish fracture as the sole driver of mortality or frailty progression.

Current anti-osteoporosis therapies have established efficacy in reducing fracture risk, but the clinical pathways built around them remain largely organised around bone remodelling and preservation of bone mass (14). In routine practice, patients of similar age and with comparable BMD or FRAX probability can differ markedly in post-fracture repair kinetics and functional recovery. This heterogeneity places skeletal fragility within a broader framework of biological ageing. Biological age can diverge from chronological age and can be quantified across multiple physiological systems (15). The hallmarks of ageing include genomic instability, epigenetic alterations, mitochondrial dysfunction, cellular senescence, stem-cell exhaustion, chronic inflammation and altered intercellular communication (16). In the immune system, impaired protective immunity coexists with low-grade chronic inflammation (17–19). This combination provides a biological route from bone marrow niche ageing to altered bone quality and fracture-repair trajectories.

**Figure 1:**
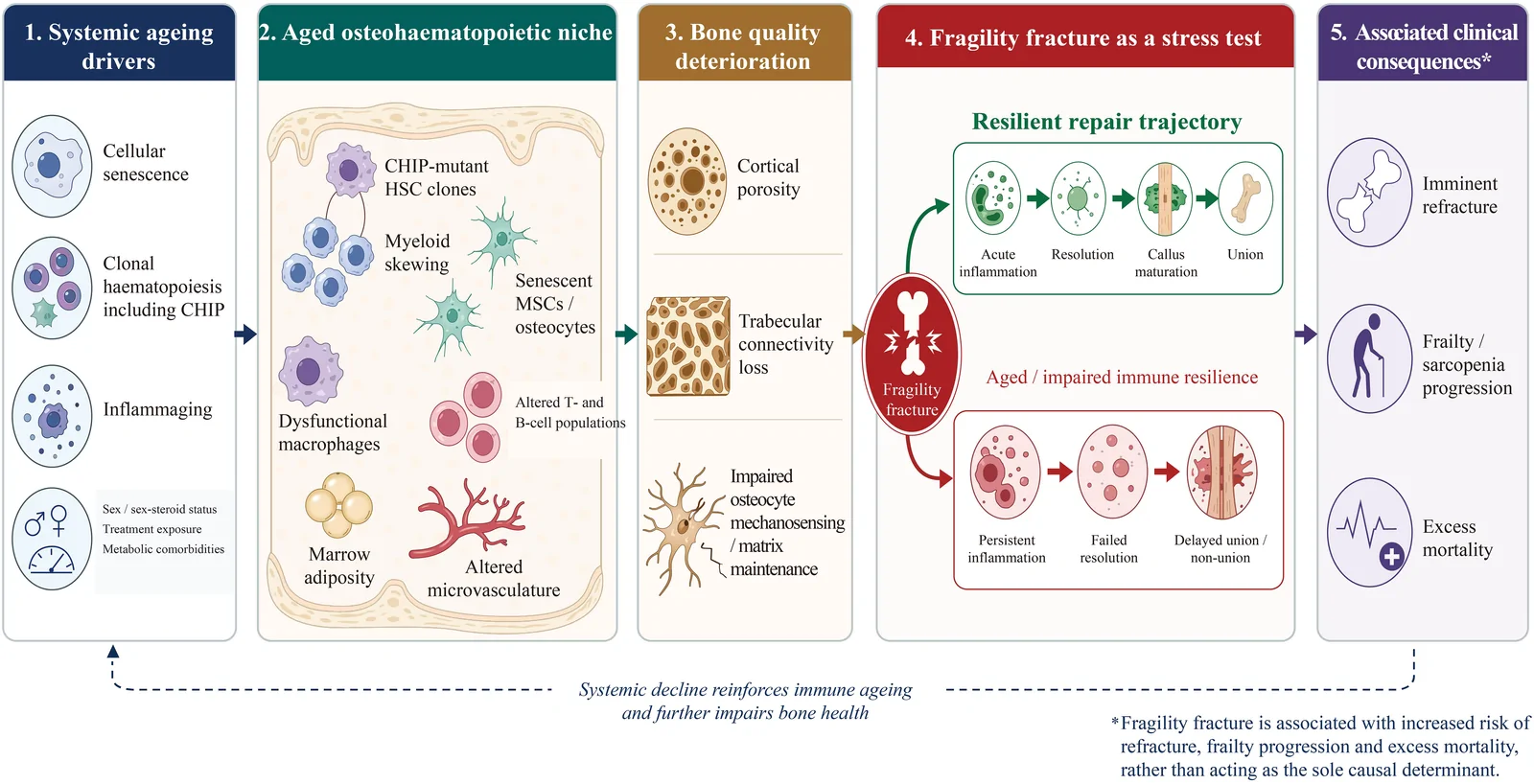
Systemic ageing drivers and host modifiers, including cellular senescence, clonal haematopoiesis of indeterminate potential (CHIP), inflammaging, biological sex/sex-steroid status, treatment exposure and metabolic comorbidities, progressively reshape the osteohaematopoietic niche. Niche dysfunction includes CHIP-mutant haematopoietic stem-cell clones, myeloid skewing, dysfunctional macrophage states, altered T- and B-cell populations, senescent mesenchymal stromal/stem cells and osteocytes, marrow adiposity and altered microvasculature. These changes may contribute to deterioration in bone quality while interacting with established endocrine, mechanical, metabolic and functional determinants. Fragility fracture is conceptualised as a stress test of immune resilience: a resilient trajectory proceeds from an appropriate acute inflammatory response through active resolution, callus maturation and union, whereas impaired immune resilience favours persistent inflammation, failed resolution and delayed union or non-union. Imminent refracture, frailty or sarcopenia progression and excess mortality are associated postfracture outcomes shaped by systemic vulnerability, comorbidities, functional reserve and care, rather than by fracture or immune ageing alone.

Frailty and sarcopenia further emphasise that skeletal fragility is not an isolated orthopaedic problem. Frailty reflects vulnerability to stressors as multisystem physiological reserve declines, whereas sarcopenia affects fall risk, mechanical loading and rehabilitation through losses of muscle strength, mass and performance (20, 21). We therefore position skeletal fragility in older adults as a composite phenotype of biological ageing within the bone-immune-haematopoietic system. **Figure 1** summarises this framework: systemic ageing remodels the osteohaematopoietic niche; niche dysfunction contributes to bone-quality deterioration alongside endocrine, mechanical, metabolic and functional determinants; and fragility fracture acts as a stress test of inflammatory resolution and tissue repair. CHIP remains a mechanistic lead for myeloid inflammatory amplification rather than a clinically validated FLS biomarker.

This review is therefore narrower than a general survey of immunosenescence in chronic bone disease. It centres on the failed transition from inflammation to repair after fragility fracture, asking whether CHIP amplifies myeloid inflammation, macrophage and regulatory T-cell programmes fail to resolve it, and persistent senescence-associated signalling maintains a non-regenerative callus and marrow niche. This fracture-phase perspective separates mechanisms associated with chronic bone loss from those that may explain delayed union and recovery.

## 2 Search approach and evidence appraisal

We developed this article as a critical narrative synthesis supported by a structured literature search. Its purpose was to integrate mechanistic, translational and clinical evidence on immune ageing, skeletal fragility and fracture repair, rather than to estimate pooled effects or meet the criteria of a systematic review.

PubMed/MEDLINE, Embase and the Web of Science Core Collection were searched on 8 May 2026. Searches in PubMed/MEDLINE were limited to records published between 1 January 2000 and 30 April 2026, whereas searches in Embase and the Web of Science Core Collection were restricted to the publication years 2000–2026. The search concepts covered immune ageing, immunosenescence, inflammaging, clonal haematopoiesis, CHIP, cellular senescence, macrophages, regulatory T cells, Th17 cells, osteoporosis, bone quality, osteoimmunology and fracture repair. Database-specific search strategies and yields are reported in **Supplementary Table 1**.

We prioritised genetic, interventional, cell-specific, lineage-tracing and temporally resolved studies when assessing mechanism. Human relevance was evaluated using cohort studies, fracture-specific clinical studies, imaging studies, biomarker analyses and recent reviews that clarified emerging concepts. Candidate biomarkers were considered clinically mature only when they showed fracture-specific validation, incremental value beyond established risk factors and practical feasibility.

Because the evidence spans animal models, observational cohorts, clinical fracture studies and omics datasets, causal language was reserved for genetic, cellular or interventional evidence. Peripheral immune measurements were not assumed to reproduce the compartmentalised biology of bone marrow or fracture callus. No formal risk-of-bias assessment, PRISMA flow diagram or quantitative synthesis was performed.

## 3 The age-altered osteoimmune niche: convergence of haematopoietic, immune and stromal ageing

### 3.1 Osteohaematopoietic niche remodelling

Bone marrow is not merely a haematopoietic compartment, but a shared niche for the skeletal, haematopoietic and immune systems. HSCs, stromal cells, vascular endothelial cells, osteocytes, osteoblasts, osteoclasts, macrophages and T cells form a dynamic intercellular communication network through direct cell-cell contact, cytokines, chemokines, EVs and metabolic signals. Osteoimmunological studies have long shown that the immune and skeletal systems share multiple regulatory axes, with osteoclastogenesis, immune-cell activation and osteogenic differentiation being closely interwoven (22).

Single-cell and spatial omics studies have shown that the adult bone marrow niche has a defined cellular and spatial architecture, rather than representing a random mixture of cell populations (23). With ageing, vascular and stromal support is progressively remodelled. Type H vessels are implicated in angiogenesis-osteogenesis coupling, and endothelial Notch signalling can influence intraosseous angiogenesis and bone formation (24, 25). Ageing perturbs HSC-niche interactions, while vascular, stromal and metabolic remodelling of the bone marrow niche becomes increasingly prominent (26). Ageing- and obesity-associated expansion of adipogenic lineages can impair osteogenic and haematopoietic regeneration, and leptin receptor-associated stromal signalling may reduce osteogenic support while increasing adipogenic bias (27, 28). These observations do not indicate a uniform decline across all aged bone marrow, but suggest that vascular, stromal and metabolic niches jointly shape the haematopoietic and bone-repair environment.

Ageing of the haematopoietic system adds a further layer of biological context. In humans, bone marrow HSCs show age-related changes in frequency and myeloid skewing, whereas clonal analyses in animals reveal multiple functional defects in aged HSCs (29, 30). Within the local bone marrow environment, such myeloid skewing may increase inflammatory monocyte-macrophage output and intersect with the osteoclast precursor lineage. Winter et al. (31) placed clonal haematopoiesis within the ageing osteohaematopoietic niche, emphasising potential bidirectional interactions between mutant haematopoietic clones and the bone marrow microenvironment.

### 3.2 CHIP-associated myeloid inflammatory amplification

CHIP is conventionally defined as clonal haematopoiesis involving a recognised myeloid driver mutation with a variant allele frequency (VAF) of at least 2%, in the absence of an overt haematological neoplasm or an otherwise explanatory cytopenia. This 2% threshold reflects historical limits of sequencing detection and nosological convention rather than an established biological threshold for skeletal effects (32–35). More sensitive sequencing can identify smaller clones, while longitudinal studies indicate that clone size and clonal expansion vary according to both the driver mutation and host context (36, 37). VAF should therefore be analysed as a measure of clone size and potential inflammatory exposure, and explored as a possible effect modifier, rather than treated merely as a nuisance covariate. Fracture studies should report sequencing depth, model VAF both continuously and within prespecified categories, and distinguish individuals carrying single from multiple clones.

Evidence varies substantially across driver mutations. TET2-deficient clonal haematopoiesis enhances macrophage NLRP3–IL-1β signalling and accelerates atherosclerosis in mice, while human CHIP is associated with increased cardiovascular risk (38, 39). Direct evidence for a TET2-driven skeletal phenotype, however, remains limited. By contrast, experimental models have shown that *Dnmt3a*-mutant clonal haematopoiesis produces an osteoporosis-like phenotype and that DNMT3A mutation-driven clonal haematopoiesis promotes inflammatory bone loss (40, 41). ASXL1 is epidemiologically important in clonal haematopoiesis, but direct evidence linking it to bone loss or fracture repair remains sparse. The available literature therefore supports a DNMT3A-specific skeletal signal, but does not permit a head-to-head conclusion that TET2 exerts a quantitatively weaker skeletal effect.

Clinical interpretation must remain outcome-specific. Evidence is strongest for DNMT3A-associated inflammatory bone loss, less developed for cortical porosity or matrix deterioration, and insufficient for predicting delayed union, non-union or functional recovery. Any association may be modified by the driver mutation, VAF, clonal growth, biological sex, metabolic state and local tissue context (36, 37). Prospective fracture cohorts should examine driver-by-VAF interactions and interactions with biological sex, while adjusting for age, BMD, previous fracture, frailty, sarcopenia, cardiovascular and renal disease, smoking, inflammation and osteoporosis-treatment history. Until such evidence becomes available, CHIP should be regarded as a mechanistic covariate rather than a decision marker for fracture liaison services.

### 3.3 Inflammaging, gut-derived signals and adaptive immune skewing

Inflammaging denotes the low-grade, chronic systemic inflammatory state that develops with advancing age (17, 42, 43). It cannot be considered separately from the acute response to fracture. Pre-existing myeloid priming, altered cytokine tone and impaired pro-resolving capacity may modify the amplitude, cellular composition and decay kinetics of the post-fracture response, increasing the likelihood that an initially necessary inflammatory phase becomes prolonged or fails to transition towards repair. Within the skeleton, inflammatory mediators regulate the RANKL-RANK-OPG axis, osteoclast differentiation, osteocytic RANKL expression and compensatory bone formation (44–47). Associations between inflammatory markers, fracture risk and frailty do not, however, establish causality, as infection, obesity, renal or metabolic disease, and poor overall health may produce similar inflammatory readouts (48–51).

The gut-immune-bone axis represents one potential upstream influence. Age-related barrier dysfunction and dysbiosis may increase exposure to microbe-associated molecular patterns, alter short-chain fatty acid and bile acid metabolism, and modify systemic and intestinal T-cell differentiation (52, 53). In mice, callus γδ T cells and microbiota-induced intestinal Th17 cells supported fracture healing, indicating that gut-derived immune responses may be reparative in some contexts rather than uniformly osteoclastogenic (54). Supplementation with *Bifidobacterium longum* also ameliorated the age-related delay in fracture repair in mice (55). These findings warrant further mechanistic investigation but do not justify clinical microbiome testing. The relevant pathways remain dependent on the experimental model and biological context, microbial signatures vary between populations, and no human fracture cohort has yet demonstrated incremental predictive value for delayed union.

Adaptive immunity also undergoes substantial remodelling with age. Thymic involution, depletion of naïve T cells, accumulation of memory or senescent T cells, and reduced T-cell receptor diversity alter the cytokine milieu of the bone marrow (18, 19, 56). Experimental osteoimmunology provides causal support for the involvement of the Th17/Treg axis: IL-23-IL-17 signalling promotes inflammatory and osteoclastogenic programmes, whereas Treg cells suppress osteoclast formation and contribute to tissue protection (57–60). Oestrogen loss can activate memory T-cell programmes that compromise bone integrity (61). Conversely, the aged stromal and myeloid environment, together with persistent SASP signalling, may itself alter T-cell differentiation. Th17/Treg imbalance should therefore be regarded as a potentially bidirectional process-both a driver and a consequence of niche dysfunction-and circulating Th17/Treg ratios should not be assumed to reflect local callus biology.

B cells constitute another component of adaptive skeletal ageing. Combined B- and T-cell deficiency impairs the attainment and maintenance of bone mass in mice, while bone marrow B cells contribute to the production of both osteoprotegerin (OPG) and RANKL (62). Ageing may increase the production of both mediators, making the net skeletal effect dependent on the B-cell subset and local tissue context (63). A recently identified RANKL^+^/CXCR4^+^ bone marrow B-cell population accumulates with age and induces bone loss in mice (64). These findings support consideration of the B-cell OPG/RANKL balance within the adaptive immune module. Direct evidence linking B-cell senescence or autoantibody-mediated mechanisms to fragility-fracture repair, however, remains insufficient. B-cell phenotypes should therefore be regarded as mechanistic candidates rather than validated biomarkers of fracture repair.

### 3.4 Biological sex as a cross-cutting modifier

Biological sex should be considered a cross-cutting modifier rather than a distinct mechanism of ageing. Menopause-associated oestrogen loss can amplify T-cell activation, RANKL signalling and bone loss (61), whereas ageing, comorbidity profiles and sex-steroid trajectories follow different patterns in men. Patterns of clonal haematopoiesis also vary by driver mutation. One population-based analysis found DNMT3A-mutant clones to be associated with female sex, whereas longitudinal data linked male sex more strongly to ASXL1 and splice-factor clones (36, 37). These findings do not support a general conclusion that CHIP burden is consistently greater in either sex.

Future fracture cohorts should prespecify sex-stratified analyses and sex-by-biomarker interaction tests for CHIP driver and VAF, inflammatory trajectories, Th17/Treg and B-cell phenotypes, and senescence-associated markers. Menopausal status, hormone therapy and other relevant endocrine treatments should also be recorded. Sex-specific biomarker thresholds or treatment recommendations remain premature until adequately powered, fracture-specific studies demonstrate meaningful differences in calibration, predictive performance or therapeutic response.

### 3.5 Cellular senescence, SASP and EV-mediated niche propagation

Cellular senescence provides a local amplification mechanism linking immune ageing to skeletal fragility. Senescent cells are characterised by stable cell-cycle arrest, DNA damage responses and resistance to apoptosis (65–67). Senescent cells can also develop the SASP, a secretory programme that mediates cell-nonautonomous inflammatory and tissue-remodelling effects (68, 69). Within the bone marrow, senescent osteocytes, mesenchymal stromal cells, immune cells, endothelial cells and macrophages can each reshape the local microenvironment. Targeting senescent cells prevents age-related bone loss in mice, and osteocytes have also been shown to regulate ageing of both bone and bone marrow (70, 71). Senescent immune cells can release grancalcin to promote skeletal ageing, whereas aged bone marrow macrophages may induce paracrine senescence through EVs (72, 73).

The evidence does not support viewing the SASP as a fixed or uniformly deleterious secretory programme. Its effects depend on the cellular source, duration, tissue context and stage of injury. Transient SASP activity may support injury responses and tissue reprogramming, whereas persistent signalling can sustain low-grade inflammation, induce bystander senescence and restrict osteogenic differentiation (74–76). Senescent cells accumulate transiently in the murine fracture callus, yet fracture-specific studies have not established that this accumulation is required for healing. In young adult mice, intermittent senolytic treatment reduced senescence-associated markers and accelerated fracture healing. In aged mice, short-term treatment with dasatinib plus quercetin depleted senescent cells within the callus and improved healing, while senescent cells derived from aged callus inhibited mesenchymal progenitors through TGF-β1 signalling (77, 78). Cellular senescence therefore remains a high-priority mechanistic target, although current evidence does not define a clinically applicable early therapeutic window (79).

CHIP and senescence may participate in a feed-forward circuit rather than operate as independent modules. Mutant myeloid cells could expose osteocytes and mesenchymal stromal cells to sustained IL-1β, IL-6, TNF-α or extracellular-vesicle cargo, thereby promoting bystander senescence. Conversely, a SASP-rich marrow niche could alter haematopoietic selection and favour the persistence or expansion of inflammatory clones (31, 38, 40, 41, 72, 73). This framework represents a testable synthesis rather than an established mechanism of fracture repair: bidirectional CHIP–senescence crosstalk has not yet been demonstrated in human fracture callus. Paired clonal genotyping and spatially resolved profiling of the callus will be needed to determine cellular directionality and temporal sequence.

Evidence maturity differs across modules. Direct skeletal CHIP evidence remains concentrated in DNMT3A models, whereas senescence and age-altered macrophage function have more direct fracture-repair support. Human inflammaging data remain associative, and adaptive immune, gut-derived and CHIP–senescence mechanisms lack phase-resolved longitudinal validation. **Table 1** summarises these outcome-specific differences.

**Table 1:** Evidence matrix linking immune-ageing mechanisms to skeletal phenotypes.

Mechanistic module	Key cells or mediators	Main skeletal phenotype	Evidence level	Major unresolved question	Key references
Driver- and VAF-specific CHIP	DNMT3A, TET2 and ASXL1 clones; clone size/growth; inflammatory monocytes/macrophages; NLRP3; IL-1β	Inflammatory osteoclastogenesis and bone loss in genetically defined subgroups; repair effect unknown	Direct bone evidence for DNMT3A; systemic inflammatory evidence for TET2; skeletal ASXL1 and human repair evidence insufficient	Do driver, VAF and clonal dynamics predict fracture or repair beyond established risks?	(31–41)
Inflammaging	CRP, IL-6, TNF-α; NLR, MLR and SII; frailty and metabolic context	Chronic priming may alter the amplitude and decay of the acute fracture response	Substantial human association evidence; limited specificity and causal inference	Which trajectories reflect causal resolution failure rather than comorbidity or trauma?	(17, 19, 42–51)
Gut–immune–bone inputs	Barrier integrity; MAMPs; SCFAs; bile acids; intestinal Th17 and γδ T cells; microbiota	Context-dependent systemic inflammation, T-cell programming and fracture repair	Mechanistic and interventional murine evidence; no validated human repair prediction	Which microbial functions are reproducible and phase-specific in older fracture patients?	(52–55)
Adaptive T- and B-cell ageing	Naïve/memory T cells; Th17/Treg; repair Treg; B-cell subsets; OPG/RANKL	Osteoclast regulation, immune restraint and progenitor support; age-related bone loss	Strong osteoimmune rationale; direct B-cell and fracture-phase human validation limited	Are local adaptive changes causes, consequences or both, and can blood capture them?	(56–64, 102, 103)
Macrophage dysfunction and resolution failure	Efferocytosis; macrophage-state continuum; SPMs (including MaR1 and RvE1); NAD^+^-linked immunometabolism	Persistent inflammation, delayed phase transition and callus maturation	Moderate-to-strong preclinical repair evidence; sparse longitudinal human evidence	Which serial local/peripheral markers identify failed resolution before delayed union?	(90, 91, 94–101, 124–127)
Cellular senescence and CHIP–senescence crosstalk	Senescent osteocytes, MSCs and immune cells; SASP; TGF-β1; grancalcin; EV cargo	Bystander senescence, suppressed osteogenesis and impaired callus maturation; proposed feed-forward interaction with CHIP	Strong cellular/animal evidence; crosstalk and human fracture validation unproven	Does failed clearance drive persistence, and does CHIP amplify it in callus?	(31, 38, 40, 41, 65–79)
Osteohaematopoietic niche and bone quality	Vascular and stromal niche; adipocytes; HSCs; osteocytes; matrix and mechanical context	Reduced regenerative reserve, cortical porosity and material deterioration beyond BMD	Strong structural and preclinical evidence; immune-specific incremental value uncertain	Which tissue and imaging readouts best calibrate immune-ageing effects?	(7–9, 23–31, 46, 80–87)

Evidence levels are outcome-specific and qualitative; they do not represent a formal risk-of-bias grade. Biological sex, age, comorbidity and osteoporosis-treatment exposure are cross-cutting modifiers. See the Abbreviations list in the main text.

## 4 Bone quality beyond BMD: organ-level expression of immune ageing

BMD remains one of the most robust and accessible quantitative measures for fracture-risk assessment, but bone strength is determined by both bone mass and bone quality. Bone quality encompasses cortical porosity, trabecular connectivity, the osteocyte lacuno-canalicular network, collagen matrix properties, mineralisation heterogeneity and microdamage repair (80–82). The organ-level readout of an aged osteoimmune niche extends beyond BMD loss to structural and material fragility. It operates alongside sex-steroid deficiency, Wnt/β-catenin and sclerostin-related signalling (83), mechanical loading, renal and metabolic disease, medication exposure and physical function.

Cortical porosity is a key phenotype of skeletal fragility in later life. Even when BMD reduction is modest, increased cortical porosity weakens the resistance of long bones and the hip to bending, torsion and fatigue damage. Bala et al. (84) showed that cortical porosity identifies women with osteopenia who are at increased risk of forearm fracture, whereas Stein et al. (85) reported abnormal trabecular plate structure and cortical thinning in postmenopausal women with osteopenia and fractures. Experimental studies link RANKL, IL-17, TNF-α, myeloid-cell skewing and senescent osteocyte signalling to osteoclastogenic and senescence pathways capable of promoting cortical porosity and loss of trabecular connectivity. These immune pathways interact with established endocrine, biomechanical and metabolic drivers of bone loss. Current clinical data cannot quantify the independent contribution of immune ageing without longitudinal imaging, treatment exposure data and careful adjustment for frailty and comorbidities.

Osteocyte senescence links immune signalling, mechanosensing and bone material quality. Osteocytic RANKL is important for age-related cortical bone loss and can be induced by senescence-associated signals (47). Ageing-related loss of osteocyte-dependent collagen maintenance and senescence-associated changes in osteocyte mechanics impair bone material quality and mechanosensation (86, 87). These findings define a cellular route through which immune ageing could produce skeletal fragility among individuals with similar BMD, although they do not directly quantify microcrack repair.

Assessing bone quality requires tools beyond DXA. Trabecular bone score (TBS) extracts textural information from DXA images, high-resolution peripheral quantitative computed tomography (HR-pQCT) quantifies cortical porosity and trabecular microstructure, and percutaneous microindentation estimates bone material strength (7–9). These approaches bridge mechanistic research and clinical stratification but have not replaced conventional risk tools. Their near-term value lies in integration with established clinical variables and validated imaging phenotypes, followed by explicit tests of whether inflammatory markers, immune-cell profiles, CHIP status, senescence-related markers and functional status add predictive information beyond existing risk models.

## 5 Defective resolution during fracture repair: fragility fracture as a stress test

Fracture repair is a temporally ordered programme involving immune, vascular, cartilaginous and bone-regenerative processes. It typically progresses through haematoma formation, acute inflammation, angiogenesis, cartilaginous callus formation, mineralisation of the hard callus and subsequent remodelling (88, 89). Successful progression through these phases requires coordination among local cytokine signals, immune-cell state transitions, mesenchymal stromal/stem cell (MSC) recruitment and the mechanical environment. The inflammatory response is required to initiate fracture healing and can influence subsequent reparative processes (90). More recent work in fracture osteoimmunology further indicates that immune-cell programmes regulate transitions between successive phases of repair (91).

Here, immune resilience refers to the capacity to mount a proportionate acute response to fracture, clear microbes, damaged cells and debris, actively resolve inflammation, and progress into reparative and remodelling states. It is a phase-resolved property rather than a single circulating biomarker. Operational assessment requires characterisation of the baseline host state and serial sampling aligned with injury and treatment. Such trajectories could be used to quantify response amplitude, time to peak, decay slope, area under the response curve and return towards baseline, and to relate these features to callus maturation and functional recovery.

Inflammatory and senescence-associated signals are not intrinsically pathological. Early inflammation and debris clearance support progenitor recruitment, angiogenesis and callus formation, while senescent cells may appear transiently during repair (70, 77, 78). We propose that a transient senescence response becomes pathological when immune clearance and macrophage efferocytosis are inadequate, allowing senescent cells and SASP signalling to persist beyond their beneficial window. CHIP-associated macrophage dysfunction could reinforce this persistence, although this link has not been demonstrated in the fracture callus. This hypothesis predicts that delayed clearance, rather than the mere detection of senescence markers, will be associated with prolonged inflammatory trajectories and delayed callus maturation.

Pre-existing inflammaging and the acute fracture response are therefore distinct but coupled processes. Baseline myeloid priming, frailty, sarcopenia and multimorbidity may increase the magnitude of the acute response, impair its resolution or delay the return towards homeostasis (20, 21, 92, 93). Specialised pro-resolving mediators regulate neutrophil trafficking, macrophage efferocytosis and tissue repair without being equivalent to non-specific immunosuppression (94, 95). Repair failure may therefore reflect excessive inflammatory input, inadequate pro-resolving signalling, defective clearance or combinations of these disturbances that vary among patients and across repair phases.

Animal studies link age-related impairment of fracture repair to disrupted inflammatory timing. In aged mouse and rat models, impaired healing is accompanied by sustained callus inflammation, reduced cellular proliferation and deficient reparative macrophage function (96–98). The conventional classically activated/alternatively activated macrophage (M1/M2) dichotomy is too restrictive to capture the range of macrophage states involved in fracture repair. Macrophages instead occupy a continuum extending from inflammatory clearance and efferocytosis/pro-resolution to reparative and remodelling states (99, 100), shaped in part by nicotinamide adenine dinucleotide (NAD^+^) metabolism and immunometabolism (101).

Treg-cell programmes provide another route through which adaptive immunity supports tissue repair. Treg cells suppress osteoclast formation and promote tissue protection and fracture repair through amphiregulin, progranulin and related mediators (59, 102, 103). Chen et al. (103) identified a CCL1-CCR8-BATF–PGRN pathway through which CCR8^+^ Treg cells support the accumulation and osteogenic differentiation of skeletal stem/progenitor cells after fracture. Whether chronic inflammaging or persistent SASP exposure impairs this pathway in the aged osteoimmune niche remains an important, testable hypothesis. Reduced Treg-cell abundance, migration, stability or reparative programming could weaken local inflammatory restraint and progenitor-cell support, while Th17/Treg skewing could shift the balance towards IL-17/RANKL-associated pro-osteoclastogenic signalling. Mechanistically, tissue-resident Treg repair programmes warrant greater priority than circulating Treg measurements or Th17/Treg ratios, which remain immature clinical predictors of fracture repair.

The most informative cohort design would combine longitudinal blood sampling with paired local tissue where feasible. Samples collected before or at presentation and during prespecified inflammatory, reparative and remodelling phases should be linked to fracture type, fixation, infection, medication exposure, radiographic callus maturation, union and function. Surgical waste tissue, marrow aspirates or revision specimens could calibrate peripheral trajectories against local states. A resilient trajectory should show a proportionate peak followed by clearance and transition to repair; a failed-resolution trajectory should show delayed decay, persistent SASP or efferocytosis abnormalities, or discordance between systemic recovery and callus maturation.

**Figure 2:**
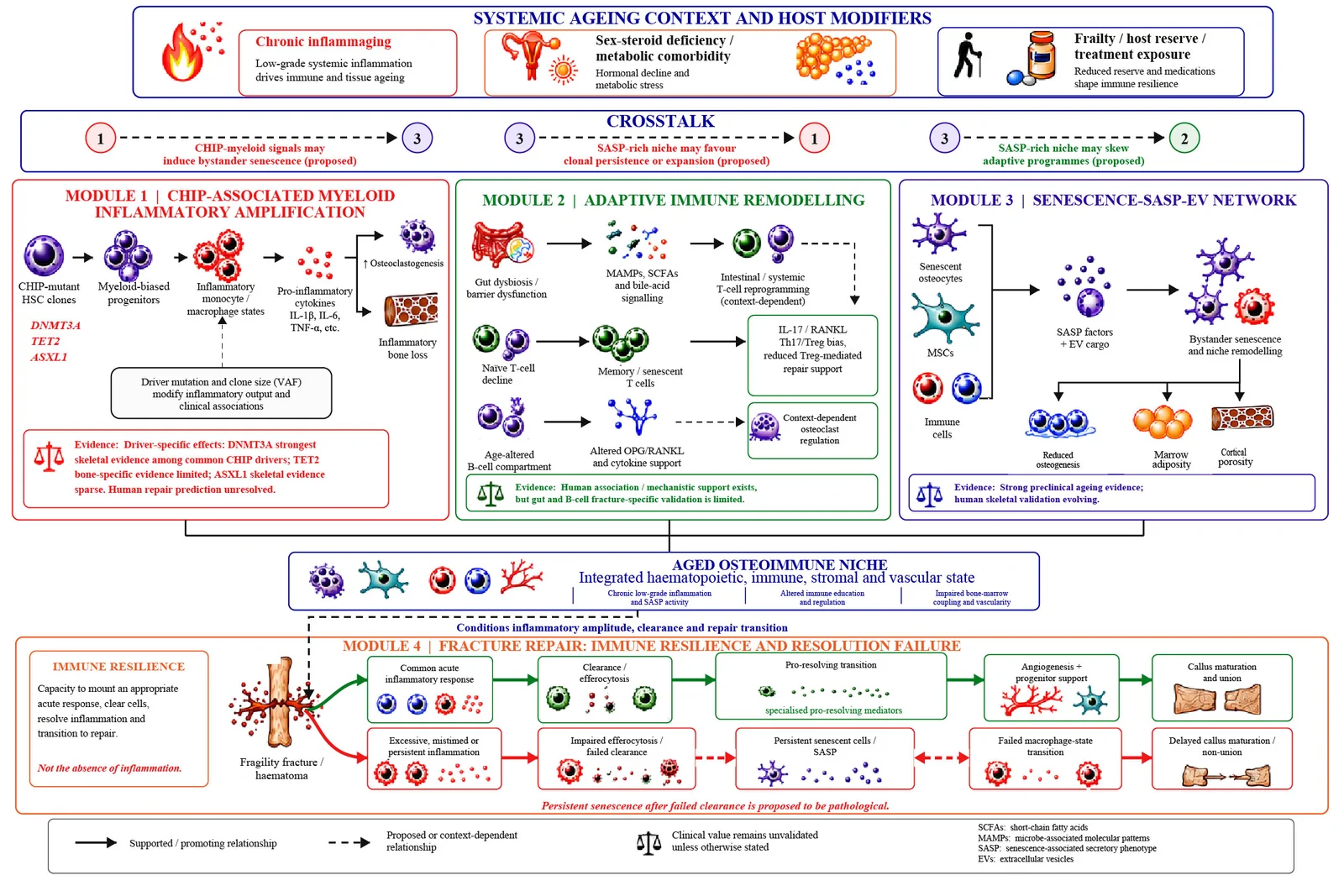
Immune-ageing mechanisms linking the aged osteoimmune niche to bone-quality deterioration and impaired fracture repair. The schematic integrates four interconnected modules within the broader context of systemic ageing, sex-steroid deficiency, metabolic comorbidity, frailty, host reserve and treatment exposure. Module 1 depicts CHIP-associated myeloid inflammatory amplification and distinguishes the uneven skeletal evidence across DNMT3A-, TET2- and ASXL1-associated clonal haematopoiesis. Variant allele frequency and clone size are shown as modifiers of inflammatory output; direct skeletal evidence is strongest for DNMT3A, limited for TET2 and sparse for ASXL1. Module 2 integrates gut-derived signals, T-cell ageing and age-altered B-cell phenotypes through context-dependent adaptive immune programming and OPG/RANKL-related regulation. Module 3 shows senescent osteocytes, mesenchymal stromal/stem cells and immune cells releasing senescence-associated secretory phenotype factors and extracellular-vesicle cargo, thereby promoting bystander senescence and remodelling of the local niche. Proposed crosstalk includes bidirectional interactions between CHIP-associated myeloid inflammation and the senescent niche, as well as SASP-related skewing of adaptive immune programmes. Modules 1–3 converge on an aged osteoimmune niche representing the integrated haematopoietic, immune, stromal and vascular state that conditions inflammatory amplitude, clearance efficiency and the transition to repair after fracture. Module 4 contrasts a resilient trajectory of proportionate acute inflammation, efferocytosis, pro-resolving transition, angiogenesis and callus maturation with an impaired trajectory characterised by excessive, mistimed or persistent inflammation, failed clearance, persistent senescence, defective macrophage-state transition and delayed union or non-union. Solid arrows denote relatively supported promoting relationships. Dashed arrows denote proposed, indirect or context-dependent relationships, including the proposed link between failed efferocytosis or clearance and persistent senescent-cell or SASP signalling, the bidirectional relationship between persistent senescence and failed macrophage-state transition, CHIP–senescence crosstalk, SASP-related adaptive immune skewing, gut-derived adaptive programming and the influence of the aged niche on the fracture-repair trajectory. Clinical predictive value remains unvalidated unless otherwise stated.

Refracture, frailty progression and mortality are convergent outcomes shaped by immune, functional, surgical and care-related factors; no single immune mechanism can account for them (20, 21, 92, 93, 104). Failure of inflammatory resolution should therefore be regarded as a testable contributor rather than a proven unifying pathway. **Figure 2** integrates CHIP-associated myeloid amplification, adaptive T- and B-cell skewing, gut-derived inputs, the senescence-SASP-EV network and macrophage resolution failure; dashed arrows denote hypotheses whose directionality or relevance to fracture repair remains unvalidated.

## 6 Biomarker maturity: from accessible inflammatory markers to spatial omics

Translating immune-ageing mechanisms into clinical tools requires explicit attention to biomarker maturity. Established clinical tools remain the foundation. Fracture-risk assessment is anchored in BMD, FRAX, prior low-trauma fracture and falls (1–6, 11, 12). Frailty and sarcopenia assessments capture functional reserve and fall propensity (20, 21), while the Strength, Assistance with walking, Rising from a chair, Climbing stairs and Falls (SARC-F) questionnaire provides a simple screen for sarcopenia risk (105). Advanced imaging and material-testing approaches provide intermediate phenotypes of bone quality, although their use remains constrained by accessibility and standardisation (7–9).

Systemic inflammatory markers, including high-sensitivity CRP (hs-CRP), IL-6, TNF-α, NLR, MLR and SII, are accessible contextual readouts of inflammaging, but they lack fracture specificity. In older fracture patients, the same markers may capture surgical stress, occult infection, renal dysfunction or frailty as much as immune ageing. Their most defensible use is therefore covariate adjustment or low-cost exploratory stratification, not stand-alone decision-making. Cellular immune profiles, including the Th17/Treg ratio, monocyte subsets, senescent T-cell populations and repair-defective Treg programmes, lie closer to mechanism. Even so, flow-cytometry panel design, sample handling, perioperative timing and fracture type can shift the result; for now these assays belong mainly in research protocols.

Senescence-associated markers and EV cargo can define aspects of a senescent niche, but they are timing-sensitive. The same SASP or EV signal may represent early repair, persistent tissue dysfunction or spillover from comorbidity, depending on cell source and sampling phase. CHIP mutations have the advantage of genomic stability; their limitation is clinical specificity. Until prospective fracture cohorts show clinically useful accuracy for fracture risk or repair outcome, CHIP status is best handled as a mechanistic covariate rather than a decision marker. Composite osteoimmunological signatures remain discovery tools (106).

The value of single-cell and spatial omics lies not only in higher resolution, but also in preservation of the anatomical and mechanical context of fracture repair. Inflammatory zones within the callus, cartilaginous callus, hard callus, angiogenic regions and cortical bone margins have distinct cellular compositions and signalling requirements. Single-cell sequencing can identify cell states, but cannot by itself determine whether those states are physically adjacent, whether they occupy high- or low-strain regions, or whether they form local communication networks. Spatial transcriptomics can resolve the proximity of senescent osteocytes, macrophages, Treg cells, MSCs and vascular cells during fracture repair, and may help determine whether resolution failure is a diffuse systemic process or a niche imbalance restricted to specific callus microregions. In bone mechanomics, spatial transcriptomic approaches can also place cellular states within local mechanical environments (107). Recent studies in mouse long-bone fracture models and complex tissues with impaired bone repair have shown that spatial omics can localise cell populations, ligand-receptor communication and local signalling defects (108, 109).

### 6.1 The challenge of compartmentalisation

A major translational constraint is that serial callus biopsies are rarely feasible in older patients with fragility fractures, particularly during the intermediate phases of repair. Spatial omics is therefore unlikely to serve as a routine *in vivo* monitoring tool, while peripheral blood markers remain imperfect surrogate readouts of a highly compartmentalised local process. The mineralised cortex, marrow cavity, fracture haematoma, developing callus vasculature and local mechanical strain and shear environment create substantial biological and spatial separation between circulating immune signals and the fracture microenvironment. Concordance between blood cytokines, cellular ratios or EV cargo and local callus transcriptomic states is therefore likely to be limited unless these peripheral measures are calibrated against tissue-resolved models.

A realistic translational pathway is necessarily stepwise. Time-resolved animal models can define the anatomical and temporal sequence of repair. Human surgical waste tissue, marrow aspirates, revision-surgery specimens and well-phenotyped non-union tissue can then be used to examine corresponding human cell states. Longitudinal blood markers, EV cargo, imaging-based measures of callus maturation and functional outcomes may subsequently be calibrated against these local reference states. The principal contribution of spatial transcriptomics may therefore be to provide anatomical and mechanical anchors for models linking local callus transcriptomic continua with peripheral readouts, including circulating EV cargo and SASP panels.

### 6.2 Clinical translation within fracture pathways

FLS and perioperative pathways provide practical platforms for biomarker translation, but the boundary between established care and biomarker discovery must remain explicit. FLS improves osteoporosis assessment, treatment initiation and secondary prevention after low-trauma fracture, while systematic reviews and meta-analyses support a reduction in subsequent fracture risk (110–113). Routine care nevertheless continues to rely on BMD, FRAX, previous fracture, falls, frailty, sarcopenia, comorbidities, medication exposure and available imaging.

**Figure 3:**
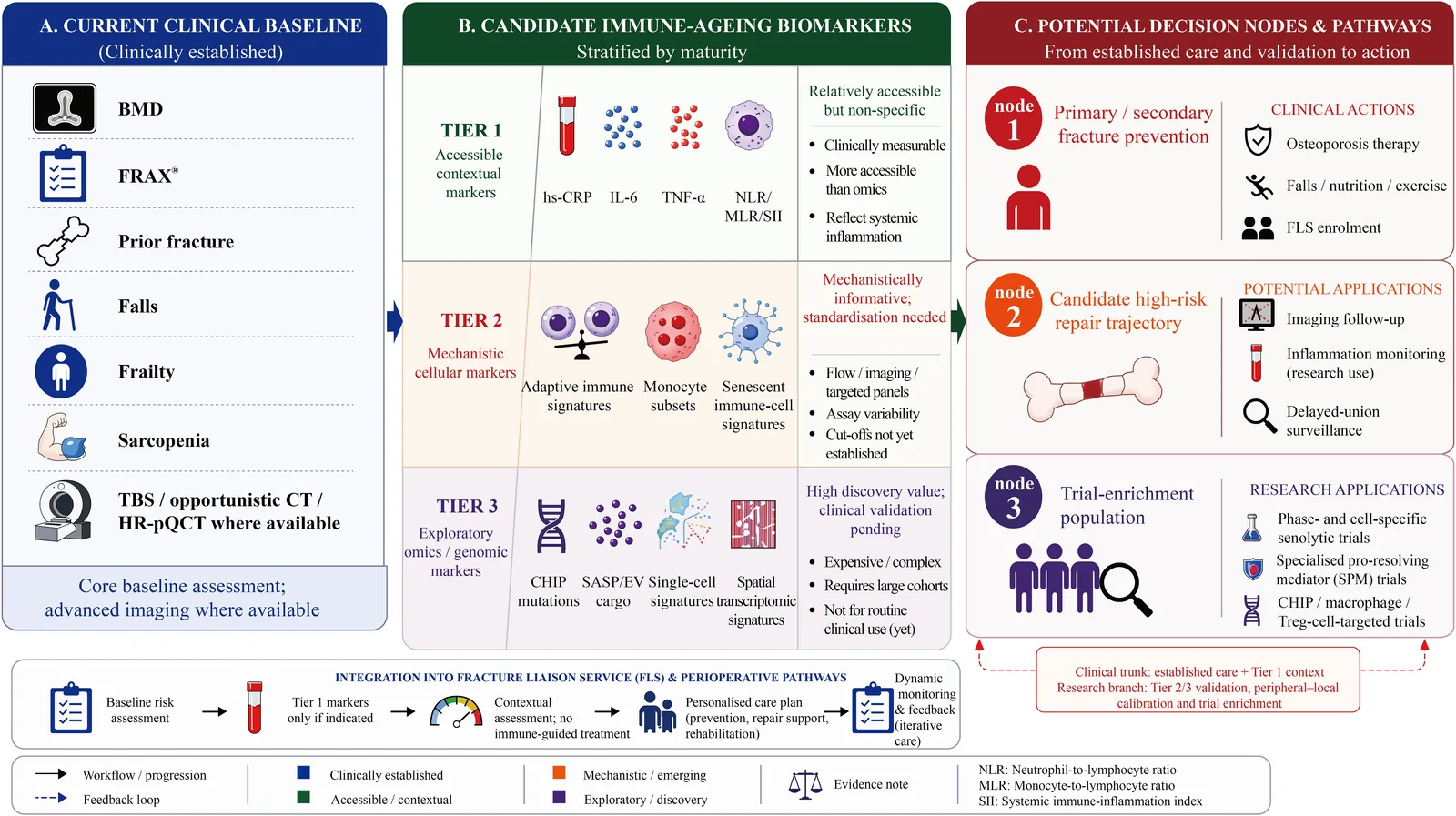
Translational roadmap for immune-ageing biomarkers and candidate skeletal geroscience pathways. Conventional fracture-risk assessment remains the clinical foundation and includes bone mineral density (BMD), the Fracture Risk Assessment Tool (FRAX), prior fracture, falls, frailty, sarcopenia and available bone-quality imaging. Candidate immune-ageing biomarkers are stratified by translational maturity. Tier 1 comprises accessible but non-specific inflammatory markers. Tier 2 includes mechanistic cellular markers, such as adaptive immune signatures, monocyte subsets and senescent immune-cell signatures. Tier 3 comprises exploratory genomic and omics markers, including clonal haematopoiesis of indeterminate potential (CHIP) mutations, senescence-associated secretory phenotype (SASP) or extracellular-vesicle cargo, and single-cell or spatial transcriptomic signatures. Peripheral biomarker readouts require calibration against local bone marrow or fracture-callus states. Routine fracture liaison service (FLS) and perioperative care remain on the established clinical pathway, whereas Tier 2 and Tier 3 markers require prospective validation and remain within research and trial-enrichment pathways. Candidate research strategies include phase- and cell-specific senolytic approaches, specialised pro-resolving mediator (SPM) trials, and CHIP-, macrophage- or regulatory T-cell-targeted programmes. None of these experimental strategies is recommended for routine fracture care without prospective validation.

Inflammatory markers may provide useful clinical context when infection, surgical stress or frailty is suspected, but they do not justify immune-guided treatment. Cellular immune markers and omics or genomic features should remain confined to embedded cohorts, enrichment protocols and validation studies until they demonstrate incremental predictive value, clinically workable turnaround times and clear implications for management. No immune-ageing biomarker currently supports routine fracture prevention or fracture-repair treatment beyond established clinical indications. **Table 2** stratifies candidate biomarkers according to category, biological meaning, feasibility, limitations and maturity, while **Figure 3** distinguishes the clinical FLS pathway from research and trial-enrichment branches.

**Table 2:** Translational maturity of skeletal immune-ageing biomarkers.

Biomarker class	Representative markers	Biological meaning	Feasibility	Major limitations	Maturity	Key references
Established clinical baseline	BMD; FRAX; prior fracture; falls; frailty/sarcopenia; treatment history; TBS, CT or HR-pQCT	Skeletal risk, functional reserve, treatment context and selected bone quality	High for core tools; variable for advanced imaging	Does not directly measure local immune resolution	Established	(1–9, 11, 12, 14, 20, 21, 92, 105)
Serial systemic inflammation	CRP, IL-6, TNF-α; NLR, MLR, SII; amplitude and decay metrics	Contextual systemic inflammatory trajectory	High to moderate	Non-specific; sensitive to trauma, infection, surgery, renal/metabolic disease and drugs	Near-translational context, not decision marker	(17, 19, 42, 43, 48–51, 104)
Cellular immune profiles	Th17/Treg; B-cell subsets and OPG/RANKL; monocytes; macrophage/efferocytosis and repair-Treg markers	Adaptive imbalance and repair-related cell states	Moderate in research settings	Gating, timing and processing variability; blood may not represent callus	Mechanistic candidate	(56–64, 99–103)
Senescence and EV measures	SASP panels; TGF-β1; grancalcin; p16/p21 signatures; EV proteins and microRNAs	Senescent burden, persistence and paracrine signalling	Low to moderate	Cell source and repair-phase ambiguity; EV methods not standardised	Exploratory/mechanistic	(65–79)
Genomic CHIP measures	Driver; VAF; clone number and growth; sequencing depth	Stable clonal exposure and myeloid ageing	Low for routine skeletal care; feasible in cohorts	No skeletal VAF threshold; driver/sex heterogeneity; repair prediction unvalidated	Clinical validation pending	(32–41)
Gut-derived measures	Microbial functions; SCFAs; barrier markers; MAMP exposure	Potential upstream immune and metabolic inputs	Low to moderate in research	Diet, antibiotic, geography and assay dependence; no human union validation	Exploratory	(52–55)
Single-cell/spatial omics	Local callus, marrow, stromal, immune and mechanical niches	Cell states, proximity and tissue compartment	Low routine feasibility; high discovery value	Tissue access, cost and reproducibility; limited serial sampling	Discovery	(23, 107–109)
Potentially feasible in structured multicentre cohorts	Clinical baseline plus serial immune trajectories, CHIP, sex, medications, local tissue calibration and healing outcomes	Tests incremental prediction and mechanism-linked enrichment	Feasible in structured multicentre cohorts	Cost and redundancy if exploratory markers lack decision impact	Future translational goal	(106–113)

Maturity refers to fracture-specific clinical use. No immune-ageing biomarker currently supports routine treatment selection. See the Abbreviations list in the main text.

## 7 Candidate geroscience opportunities and clinical constraints

The immune-ageing framework yields testable interventions for skeletal fragility and repair failure, not established therapies. Patients in routine practice are often receiving, or have previously received, standard anti-osteoporosis treatment. Every geroscience intervention must be studied within this standard-of-care context, where existing therapies act as both clinical confounders and biological modifiers of the repair niche.

Standard anti-osteoporosis therapies may influence both skeletal biology and immune readouts. Bisphosphonates inhibit osteoclast function through the mevalonate pathway. In mice, zoledronic acid also inhibits protein prenylation in alveolar and peritoneal macrophages, although comparable effects in bone marrow or fracture-callus macrophages have not been established (114). Denosumab inhibits RANKL-RANK signalling, a pathway shared by osteoclasts and immune cells. A meta-analysis reported an increased risk of serious infection-related adverse events at doses used for osteoporosis, whereas the overall risk of infection and infection-related mortality was not increased (115). By contrast, teriparatide and romosozumab have well-established skeletal effects (116, 117), but the available evidence does not establish direct effects on immune ageing or callus-resolution programmes. Biomarker studies should therefore record drug class, cumulative treatment duration, timing of the most recent dose, adherence, treatment discontinuation and potential rebound periods, sequential therapy, and timing relative to fracture and sampling. These exposures should be captured as potential confounders and biological effect modifiers, using class- and time-specific variables rather than a single binary indicator of treatment.

Senolytics and senomorphics have the clearest experimental connection to skeletal ageing, but fracture-specific timing and target-cell specificity remain unresolved. In non-skeletal settings, SASP signalling can support tissue plasticity, whereas senescent-cell clearance improves selected age-related functional and bone-loss phenotypes; human evidence remains preliminary (70, 118–121). In fracture models, intermittent senolytic treatment accelerated healing in young adult and aged mice, and distinct senescent-cell populations had different effects (77–79). These findings do not support a fixed 0-7-day avoidance window or direct extrapolation to human fracture timing. Senolytic timing should also not be conflated with non-specific early anti-inflammatory suppression. Translation requires phase- and cell-specific target engagement while preserving host defence and monitoring callus maturation, infection and wound outcomes.

Mechanistic links between CHIP, inflammaging and inflammatory amplification identify the NLRP3–IL-1β axis as a potential therapeutic target. CANTOS demonstrated that IL-1β-targeted therapy reduced recurrent cardiovascular events, but these findings cannot be extrapolated directly to fracture healing (122). Although the NLRP3 inflammasome is increasingly recognised as a druggable target in inflammatory diseases, fracture-specific evidence for its efficacy and safety remains lacking (123).

### 7.1 Specialised pro-resolving mediator-based therapy

Specialised pro-resolving mediators (SPMs), including resolvins, maresins and protectins, are conceptually attractive because they engage active programmes of inflammatory resolution and clearance rather than broadly suppressing the initiation of inflammation (94, 95, 124, 125). Maresin 1 and resolvin E1 enhanced the regenerative properties of periodontal ligament stem cells under inflammatory conditions (126), whereas protectin DX reduced fracture-induced postoperative pain in mice and enhanced GPR37-dependent macrophage efferocytosis (127). These findings support the biological plausibility of resolution-directed therapy, but neither study demonstrated improved fracture union or established efficacy in aged models of fragility fracture. SPMs should therefore remain within mechanism-led preclinical and early-phase translational pathways. Pharmacokinetics, infection and wound safety, pain, callus maturation and fracture union should be evaluated as distinct outcomes.

### 7.2 Gut-targeted strategies and trial design

Gut-targeted interventions remain experimental. Murine fracture studies involving microbiota-induced intestinal Th17 cells and *Bifidobacterium longum* suggest that microbiome effects may be beneficial in a context- and phase-dependent manner (54, 55). However, treatment responses may vary with microbial strain, diet, antibiotic exposure, geography and baseline microbiota. Probiotics, prebiotics, short-chain fatty acid-based approaches and barrier-directed therapies should not enter routine fracture care without replication in aged models, clearly defined mechanisms, and human trials establishing safety and efficacy.

Markers of Treg-cell repair programmes and macrophage states may be better suited to trial enrichment than to immediate clinical decision-making, while direct modulation of these pathways remains an experimental therapeutic strategy. A defensible trial design should integrate skeletal phenotype, functional reserve, biological sex, treatment exposure and a limited number of mechanism-linked variables. Pain should be assessed separately from radiographic callus maturation, delayed union, non-union, functional recovery, refracture, infection and wound complications. One focused experimental approach would be to determine whether interrupting persistent EV-mediated senescence signalling from aged bone marrow macrophages reduces paracrine senescence without inducing broad systemic immunosuppression (73).

## 8 Discussion and conclusions

Skeletal fragility in older adults is a composite phenotype of biological ageing expressed within the skeletal system. Direct skeletal evidence for clonal haematopoiesis is currently strongest for DNMT3A-associated inflammatory bone loss, whereas cellular senescence and macrophage dysfunction have more direct preclinical links to fracture repair. Evidence for TET2 remains predominantly systemic, skeletal data for ASXL1 are sparse, and the ability of CHIP to predict delayed union has not been validated. Age-related changes in adaptive T- and B-cell compartments, gut-derived signals and defective inflammatory resolution are biologically plausible, but require confirmation in phase-resolved human studies. Several outstanding questions arising from this framework are summarised in **Box 1**.

Box 1 **Outstanding questions in skeletal immune ageing.**

- Do CHIP driver identity, variant allele frequency (VAF), and clonal dynamics predict fracture risk or repair outcomes beyond age, bone mineral density (BMD), frailty, comorbidities, and treatment exposure?
- Can immune resilience be operationalised using phase-resolved trajectories that connect local callus states with circulating markers and clinical outcomes?
- Does bidirectional crosstalk occur within the fracture callus between CHIP-mutant myeloid cells and senescent stromal or skeletal cells?
- Can inflammatory resolution be restored safely, and is the therapeutic response modified by biological sex, gut-derived signals, or osteoporosis treatment?
- Can defective inflammatory resolution be therapeutically corrected in older patients without increasing the risks of infection, wound complications, or other immune-related adverse outcomes?

Studies addressing these questions must explicitly account for age, frailty, multimorbidity, cardiovascular and renal disease, functional reserve, fracture and surgical characteristics, biological sex, and exposure to osteoporosis treatment. In analyses of CHIP, driver identity and VAF should be modelled explicitly rather than reduced to a binary exposure. For dynamic immune markers, both the biological phase and tissue compartment must be specified.

The age-altered osteoimmune niche helps explain why patients with similar BMD or FRAX probabilities may differ in bone quality, repair kinetics and functional recovery. It extends rather than replaces conventional osteoporosis management, and its immediate value is to define testable relationships—particularly driver- and VAF-specific CHIP, failed efferocytosis and persistent senescent-cell signalling—not to justify immune-guided treatment.

Biological sex should be prespecified as a potential modifier rather than treated solely as an adjustment variable. Future fracture cohorts should test sex-by-biomarker interactions for CHIP driver and VAF, inflammatory trajectories and senescence-associated signals, and should assess model calibration separately in women and men. Menopausal status, hormone therapy and other relevant endocrine treatments should also be recorded. Sex-specific biomarker thresholds or therapeutic recommendations remain premature until adequately powered fracture studies demonstrate reproducible differences in predictive performance, target engagement or treatment response.

This critical narrative synthesis has several limitations. Although the search was structured, it was not systematic; duplicate screening, formal risk-of-bias assessment, a PRISMA flow diagram and quantitative synthesis were not undertaken. The evidence spans species, age groups, fracture sites, fixation methods and sampling phases, and positive mechanistic findings may be preferentially published. Human callus data remain scarce, peripheral measurements may not reflect tissue states, and confounding by medication exposure and comorbidity is substantial. These constraints limit causal interpretation and clinical translation, even when individual mechanisms are experimentally compelling.

The field should establish predictive validity before advancing to intervention. Prospective fracture cohorts should determine whether driver- and VAF-specific CHIP, phase-resolved immune trajectories or senescence-associated signals provide incremental information beyond BMD, bone quality, frailty, sarcopenia, biological sex, treatment exposure and fracture characteristics. Only markers with reproducible temporal profiles, incremental predictive value and clear implications for clinical management should progress to interventional trials. Such trials must assess target engagement, fracture union, functional recovery, infection and wound safety as distinct outcomes.
