# Supplementary material for: Immune ageing in skeletal fragility: clonal haematopoiesis and failure of inflammatory resolution during fracture repair
Source: Front Immunol. 2026 Aug 17;17:1913480. doi: 10.3389/fimmu.2026.1913480 (PMC13527206; doi:10.3389/fimmu.2026.1913480)
Supplement: Supplementary file 1 [file Table1.docx]

**Supplementary Table 1. Database-specific search strategies and search yield**

| **Database/search** | **Fields** | **Limits** | **Date** | **Records** |
| --- | --- | --- | --- | --- |
| PubMed/MEDLINE | MeSH + title/abstract | 1 Jan 2000–30 Apr 2026 | 8 May 2026 | 926 |
| Embase | Title/abstract | 2000–2026 | 8 May 2026 | 581 |
| Web of Science Core Collection | Topic search | 2000–2026 | 8 May 2026 | 885 |

Note. The database searches were conducted on 8 May 2026; the reported yields are those obtained on that date. The Embase strategy used title/abstract syntax; no additional Emtree expansion is claimed.

**PubMed/MEDLINE (926 records)**

(("Immunosenescence"[Mesh] OR "Cellular Senescence"[Mesh] OR "Clonal Hematopoiesis"[Mesh] OR "immune aging"[tiab] OR "immune ageing"[tiab] OR "immunosenescence"[tiab] OR "inflammaging"[tiab] OR "geroscience"[tiab] OR "clonal hematopoiesis"[tiab] OR "clonal haematopoiesis"[tiab] OR "CHIP"[tiab] OR "cellular senescence"[tiab] OR "SASP"[tiab] OR "senescence-associated secretory phenotype"[tiab] OR "aged macrophage"[tiab] OR "aged macrophages"[tiab] OR "aging macrophage"[tiab] OR "aging macrophages"[tiab] OR "ageing macrophage"[tiab] OR "ageing macrophages"[tiab] OR "senescent macrophage"[tiab] OR "senescent macrophages"[tiab] OR "aged T cell"[tiab] OR "aged T cells"[tiab] OR "senescent T cell"[tiab] OR "senescent T cells"[tiab] OR "aged Treg"[tiab] OR "aged Tregs"[tiab] OR "senescent Treg"[tiab] OR "senescent Tregs"[tiab])) AND (("Osteoporosis"[Mesh] OR "Fracture Healing"[Mesh] OR "skeletal fragility"[tiab] OR "osteoporosis"[tiab] OR "bone quality"[tiab] OR "fracture healing"[tiab] OR "fracture repair"[tiab] OR "osteoimmune"[tiab] OR "osteoimmunology"[tiab] OR "osteohaematopoietic niche"[tiab] OR "osteohematopoietic niche"[tiab] OR "bone marrow niche"[tiab])) AND ("2000/01/01"[Date - Publication] : "2026/04/30"[Date - Publication])

**Embase (581 records)**

("immune aging":ti,ab OR "immune ageing":ti,ab OR "immunosenescence":ti,ab OR "inflammaging":ti,ab OR "geroscience":ti,ab OR "clonal hematopoiesis":ti,ab OR "clonal haematopoiesis":ti,ab OR "cellular senescence":ti,ab OR "SASP":ti,ab OR ((macrophage* OR Treg* OR Th17 OR "T cell*") NEAR/3 (aging OR ageing OR aged OR senescent OR senile)):ti,ab) AND ("skeletal fragility":ti,ab OR "osteoporosis":ti,ab OR "bone quality":ti,ab OR "fracture healing":ti,ab OR "fracture repair":ti,ab OR "osteoimmune":ti,ab OR "osteoimmunology":ti,ab OR "osteohaematopoietic niche":ti,ab OR "osteohematopoietic niche":ti,ab) AND [2000-2026]/py

**Web of Science Core Collection (885 records)**

(TS=("immune ag*ing" OR "immunosenescence" OR "inflammaging" OR "geroscience" OR "clonal h*ematopoiesis" OR "CHIP" OR "cellular senescence" OR "SASP" OR ((macrophage* OR Treg* OR Th17 OR "T cell*") NEAR/3 (ag*ing OR aged OR senescent OR senile)))) AND TS=("skeletal fragility" OR "osteoporosis" OR "bone quality" OR "fractur* heal*" OR "fractur* repair*" OR "osteoimmune*" OR "osteoh*ematopoietic niche*" OR "bone marrow niche*") AND PY=(2000-2026)
